# Letter to the editor: ‘The FHJ debate: sustainable healthcare should be the responsibility of every physician’

**DOI:** 10.1016/j.fhj.2025.100449

**Published:** 2025-06-30

**Authors:** Kayan Vekaria, Berenice Langdon, Pippa Oakeshott

**Affiliations:** Population Health Research Institute, School of Health and Medical Sciences, Tooting campus, City St George's, University of London, Cranmer Terrace, London SW17 0RE, United Kingdom

Dear editor,

We agree with Thomas Daniels that almost every action that improves the environmental sustainability of healthcare also benefits patients.^1^ In line with the Royal College of Physicians’ *Green physician toolkit* https://www.rcp.ac.uk/policy-and-campaigns/policy-documents/green-physician-toolkit/, this includes preventing overprescribing and unnecessary blood tests, avoiding overuse of antibiotics, encouraging walking, cycling and travelling ‘more wisely’, moving to plant-based diets and buying less.^2^

Another possible way that healthcare professionals could cut their own carbon footprint is by reducing their use of non-sterile plastic gloves.^3^ Glove overuse can have unseen clinical and environmental consequences.

Non-sterile gloves only need to be worn when we are going to come into contact with a bodily fluid, non-intact skin or mucous membrane.^3^ Otherwise, routine clinical hand washing and/or alcohol gel should be adequate. Around 1.4 billion gloves are used across the NHS each year. By educating staff on reducing unnecessary glove use, Great Ormond Street Hospital saved 21 tonnes of plastic in 1 year,^3^ cutting both NHS costs and pollution from plastics.

Inappropriate glove use can also spread infection. This is because prolonged glove wearing can act as a breeding ground for any bacteria on the hands of the healthcare worker prior to wearing gloves. One study found that hand hygiene was not performed after removing gloves in over 40 % of cases,^4^ as many healthcare workers mistakenly believed that gloves keep their hands clean. The risk of spreading infection is exacerbated if they then touch other surfaces in the ward or clinic and/or treat patients. Healthcare workers may also use alcohol gel on gloves instead of changing them, prioritising speed over best practice.^5^

Health professionals have a key role in driving change. This could include unpacking the clinical and climate cost of inappropriate glove use. Meanwhile, global warming due to increasing greenhouse gas emissions threatens the health and survival of everyone alive today.^2^ We agree that ‘Sustainable healthcare should be the responsibility of every physician’.^1^ As well as implementing some of the measures mentioned above, we hope that health professionals will consider reducing inappropriate glove use.

## Funding

This research did not receive any specific grant from funding agencies in the public, commercial or not-for-profit sectors

## CRediT authorship contribution statement

**Kayan Vekaria:** Writing – review & editing, Writing – original draft, Conceptualization. **Berenice Langdon:** Writing – review & editing. **Pippa Oakeshott:** Writing – review & editing, Writing – original draft.

## Declaration of competing interest

The authors declare that they have no known competing financial interests or personal relationships that could have appeared to influence the work reported in this paper.
